# Anthropogenic impacts on Costa Rican bat parasitism are sex specific

**DOI:** 10.1002/ece3.2245

**Published:** 2016-06-21

**Authors:** Hannah K. Frank, Chase D. Mendenhall, Seth D. Judson, Gretchen C. Daily, Elizabeth A. Hadly

**Affiliations:** ^1^ Department of Biology Stanford University Stanford California; ^2^ Center for Conservation Biology Department of Biology Stanford University Stanford California; ^3^ David Geffen School of Medicine University of California Los Angeles California; ^4^ Stanford Woods Institute for the Environment Stanford California; ^5^ The Nature Conservancy Arlington Virginia; ^6^ Center for Innovation in Global Health Stanford University Stanford California

**Keywords:** Bat fly, conservation, dilution effect, disease, fragmentation, land‐use change

## Abstract

While anthropogenic impacts on parasitism of wildlife are receiving growing attention, whether these impacts vary in a sex‐specific manner remains little explored. Differences between the sexes in the effect of parasites, linked to anthropogenic activity, could lead to uneven sex ratios and higher population endangerment. We sampled 1108 individual bats in 18 different sites across an agricultural mosaic landscape in southern Costa Rica to investigate the relationships between anthropogenic impacts (deforestation and reductions in host species richness) and bat fly ectoparasitism of 35 species of Neotropical bats. Although female and male bat assemblages were similar across the deforestation gradient, bat fly assemblages tracked their hosts closely only on female bats. We found that in female hosts, parasite abundance per bat *decreased* with increasing bat species richness, while in male hosts, parasite abundance *increased*. We hypothesize the differences in the parasite–disturbance relationship are due to differences in roosting behavior between the sexes. We report a sex‐specific parasite–disturbance relationship and argue that sex differences in anthropogenic impacts on wildlife parasitism could impact long‐term population health and survival.

## Introduction

Humans are altering the world at an unprecedented rate, modifying habitats and disrupting relationships between coevolved species (Barnosky et al. 2012). These changes have generated interest in understanding how human actions will impact such coevolutionary relationships, including parasite dynamics in wildlife populations, as organisms that cause disease can have major conservation implications (e.g., Salkeld et al. 2013; Young et al. 2013). While there has been a great focus on disease organisms and endoparasites (e.g., Ezenwa et al. 2006; Cottontail et al. 2009; Young et al. 2013), less effort has been devoted to understanding the impact of human actions on ectoparasitism (Pilosof et al. 2012), even though ectoparasites can affect host survival and fitness, as well as vector diseases (Lehmann 1993; Allan et al. 2003). Additionally, in studies of the impacts of humans on parasite risk in natural populations, broadly applicable patterns are elusive, likely because parasite and disease risk depend on numerous subtle ecological factors that are difficult to identify in large community‐level studies (e.g., Salkeld et al. 2013). In particular, researchers often fail to consider sex‐specific differences when investigating how factors such as disturbance or biodiversity change will modify wildlife parasite dynamics.

Considering sex in the relationship between parasitism and disturbance is important because sexes often differ in numerous fundamental aspects of their biology including behavior, habitat use and longevity, as well as their respective importance for population, or species, survival (e.g., Sukumar 1991; Main et al. 1996; Rubin and Bleich 2005). Differences in spatial aggregation, social interactions or hormones could lead to greater exposure or susceptibility to parasites in one sex than the other (Zuk and McKean 1996; Rubin and Bleich 2005), and in many mammals, the sexes routinely differ in their parasite burdens (e.g., Poulin 1996; Zuk and McKean 1996; Patterson et al. 2008). If one sex is disproportionately affected by parasites and any parasite‐related disease or fitness effects, differential mortality could result in uneven sex ratios and lead to higher population extinction risk (Wedekind 2012). However, the effects of sex ratio biases depend on the biology of the organisms. For example, polygynous populations are more robust to the loss of males than to the loss of females because a single male may mate with multiple females (Sukumar 1991). Examinations of populations of species as a whole, without consideration of sex, could compromise our ability to predict the stability of such populations if the sexes constituting them are operating radically differently.

Like many mammals, male and female bats often behave differently and exhibit sexual segregation that may affect their parasite risk. In many tropical bat species (and the majority of the bat species in our study), females roost in groups in resource‐rich habitats with other females and few to no males (McCracken and Wilkinson 2000; Altringham 2011). Sexual segregation in many species of bats is particularly pronounced during the maternity season when females congregate to give birth and raise offspring, often living in larger groups and staying more faithful to their roosts than during nonbreeding periods (Fleming 1988; McCracken and Wilkinson 2000; Kunz and Lumsden 2003; Chaverri et al. 2007). In contrast, many males frequently roost singly or in low numbers away from females, especially during the nursing season because lactating females require so many resources (McCracken and Wilkinson 2000; Altringham 2011), and can display lower roost fidelity than females (e.g., Morrison 1979). Some males, however, roost with groups of females in harems year round and may therefore experience the same parasite dynamics as females (McCracken and Wilkinson 2000). Roosting habits heavily influence parasitism in bats; because blood‐sucking, host‐specific, bat fly pupae develop on roost walls before colonizing bat hosts, bats that use roosts that persist for longer and are more protected have more parasites (Dick and Patterson 2007; Patterson et al. 2007), while those that switch roosts frequently may have reduced parasitism (Lewis 1995). If human action alters roost or food availability through deforestation, it could lead to altered parasitism (Pilosof et al. 2012).

Here, we test the sex‐specific effects of human‐induced habitat conversion on ectoparasitism in 35 species of bats in an agricultural landscape in southern Costa Rica. We use tree cover and bat species richness as metrics of human influence, as other researchers have focused on disturbance and changes in biodiversity when evaluating the effect of humans on parasite or disease risk. (Some studies assume that environmental disturbance will lead to reduced biodiversity (Young et al. 2013), although this is not always the case, and indeed, sometimes bat diversity is higher in disturbed habitats [e.g., Cisneros et al. 2015].). We seek to answer three questions about how bats and their parasites vary with human influence. First, do the compositions of the male and female bat assemblages shift in a similar manner along a deforestation gradient? Second, within each sex, do changes in host assemblage composition affect parasite assemblage composition? Third, do parasite loads differ between male and female bats and do they increase with decreased host species richness or decreased tree cover in both male and female bats?

Given the host specificity of bat fly species, we predict that the parasite assemblages will track their host assemblages, but sex‐specific differences in roosting behavior may lead to different relationships between female hosts and their parasites than the majority of male hosts and their parasites.

## Methods

### Study region and sites

**Figure 1:**
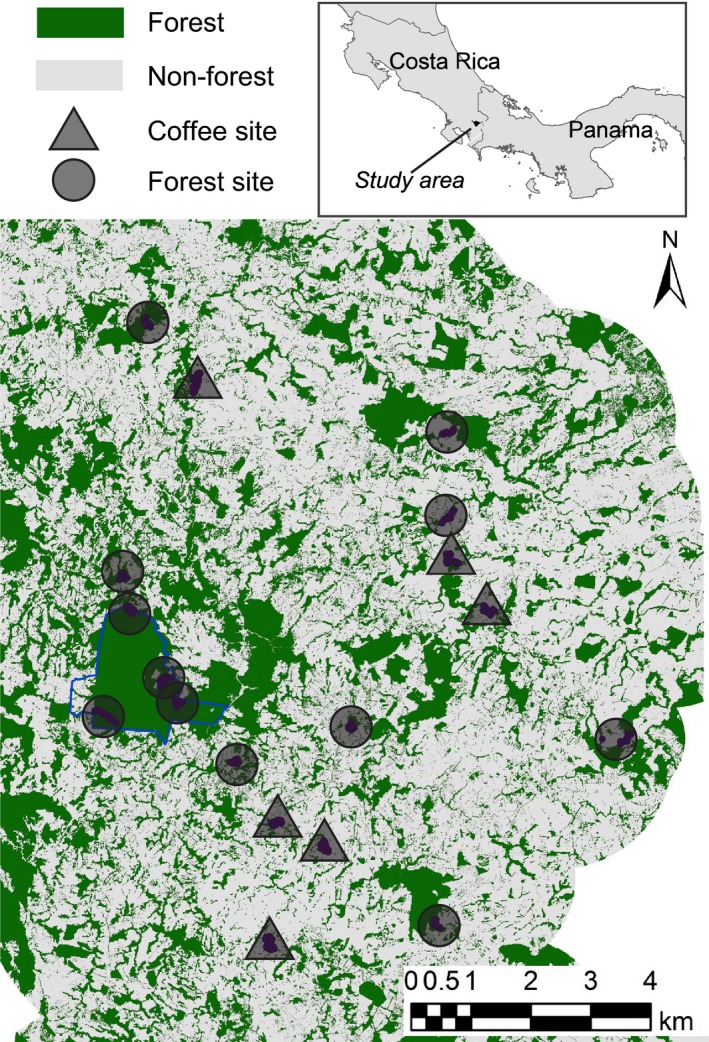
Study sites in the Costa Rican landscape. Location of study landscape is indicated on the map in the top right. Circles indicate forested sites; triangles indicate coffee sites. Dark points within sites indicate mist net locations, placed haphazardly within each site. Blue outline indicates the border of the Las Cruces Forest Reserve.

Bats were sampled in southern Costa Rica in the area around the Las Cruces Biological Station (8° 47′ N, 82° 57′ W, 1100 m). Protecting roughly 280 ha of primary and mature secondary premontane tropical wet forest (Holdridge 1967), the Las Cruces Biological Reserve lies in the Coto Brus Valley surrounded largely by pasture, cropland, houses, and remnant forest fragments (Mendenhall et al. 2014). Bats were sampled in 18 sites of varying tree cover; 12 forested sites included riparian remnant forests, small forest fragments, secondary forest, and forest reserve sites (~25–77% tree cover at a 1000 m radius). The remaining six were located in coffee plantations with ~5–25% local tree cover on the farms, described in Belisle et al. (2014). Located within a 4300‐ha area, sites were on average 5.0 km away from one another (SE = 186 m; Fig. 1). Such a landscape scale is consistent with other studies (e.g., Meyer and Kalko 2008) and known distances between roost and foraging areas (600–800 m, *Sturnira hondurensis*, Cortés‐Delgado and Sosa 2014; 800 m, *Carollia perspicillata*, Heithaus and Fleming 1978), home range sizes (9.0 ha, *Artibeus watsoni*; 3.8 ha, *Micronycteris microtis*, Albrecht et al. 2007), and feeding areas (1.5–51 ha, *Glossophaga soricina*, Lemke 1984) of many of the species we encounter.

### Bat sampling

Bats were captured at each site for one night a year between 11 February and 9 March in both 2012 and 2013 (a total of 17,280 mist net‐meter‐hours per year); data on 2012 bat captures (*N* = 469) were also included in Mendenhall et al. (2014). We used 20 ground‐level mist nets (12 m × 2.5 m, 32‐mm mesh) distributed in a 3‐ to 5‐ha plot at each site and sampled for four hours, starting at sunset, and had two to four individuals monitoring nets (Mendenhall et al. 2014). Our goal was to capture how human land use may be changing parasitism dynamics across the landscape during a season in which males and females were likely to have the greatest difference in behavior, that is, during the dry season when females of some species would be forming maternity colonies away from males. Accordingly, sites were sampled as close together temporally as possible and at the same time in both years. Additionally, three previous years of capture in these sites demonstrated that capture rates precipitously decline after the first night (data not shown), as has been observed in other systems (Marques et al. 2013). Studies which aim to characterize the bat communities across a landscape often survey sites more times over a longer period of time (e.g., Meyer and Kalko 2008; Klingbeil and Willig 2010; Cisneros et al. 2015). Our goal was to sample as temporally restricted a sample as possible and to assess parasite differences across the landscape, not to exhaustively inventory the bat assemblages. Therefore, we focused on single night, high effort surveys and may have missed a few rare species. However, we attempted to ensure that our sample faithfully reproduced known differences across sites and that we adequately sampled our localities (see below).

Captured bats were identified according to LaVal and Rodríguez‐H (2002), Reid (2009) and H. York (pers. comm.). Two of the species analyzed, *Artibeus watsoni* and *Artibeus phaeotis*, are distinguished by the presence of a molar that is difficult to observe in the field. When this molar was observed, the bat was identified as *A. watsoni*; otherwise, the bat was recorded as *A. phaeotis/watsoni*. Lumping and/or splitting these species did not qualitatively alter any results. Age was determined by examining the degree of ossification of the phalanges. In order to identify within‐year recaptures, we marked bats with collars (2012; only bats >5 g) or using wing punches (2013).

To avoid cross‐contamination of parasites between individuals, individual bats were kept in sterilized cotton bags until processing. All bats were released onsite shortly after identification and ectoparasite collection.

### Ectoparasite collection and identification

Ectoparasites were removed from individual bats using forceps and placed in 95% ethanol. Following a standardized search pattern on each individual, we counted and attempted to catch all bat flies and recorded the observed number. As our goal was to maximize the welfare of the bat by minimizing handling time and because bats in 2012 were involved in a larger mark–recapture study (Mendenhall et al. 2014), we opted not to fumigate the bats to remove ectoparasites, but rather to remove ectoparasites manually, as others have done (ter Hofstede and Fenton 2005). We were unable to exhaustively collect every fly we observed on each individual, and as such bat fly diversity could have been underestimated. However, as we followed a systematic field technique, any error should be standardized across all individuals. To ensure that we were not getting better at detecting bat flies as sampling progressed, we tested whether we detected more parasites in the last four sites in each year than the first four sites for species found in both sets.

Flies were identified morphologically by three separate observers using a stereozoom microscope. One observer (SDJ) examined a subset of flies identified by the other two observers to ensure consistency in identification. Identifications were made using published literature and guides (Wenzel et al. 1966; Miller and Tschapka 2009; Brown et al. 2010).

In a limited number of cases (6 of 1038), bat flies were found on bat species that were not previously identified as hosts by Wenzel et al. (1966). These instances were attributed to a “disturbance transfer,” which can occur when multiple bats are captured in a mist net (Wenzel et al. 1966). These few anomalous individuals were excluded from our analyses, as is standard.

### Statistical analyses

All adult bats for which we had data on parasitism, sex, roost duration (explained below), and capture location were analyzed. When bats were caught more than once within the same field season (*N* = 6), only the first observation was analyzed. All observations (excluding within‐year recaptures) from both capture years were pooled to estimate species richness in each site. The closely related fly species *Trichobius caecus* and *T. johnsonae* are hard to distinguish morphologically. In the assemblage analyses, they were treated as separate species; combining the species did not qualitatively alter our results. All statistics were performed in R (R Core Team 2014).

### Assemblage analyses

Chao dissimilarity indices between sites were calculated separately for female bats, male bats, flies on female bats, and flies on male bats. Chao dissimilarity indices were used because they can incorporate abundance data to account for the effect of unseen species and may be more accurate than the presence–absence‐based dissimilarity metrics (Chao et al. 2005). Only the flies collected off the analyzed bats were included in the assemblage analyses. No flies were found on female bats at one site; assemblage data with and without this site were analyzed to ensure this did not influence our conclusions.

To determine whether tree cover (Mendenhall et al. 2014) was correlated with each assemblage of bats or flies, a permutational multivariate analysis of variance using distance matrices with 10,000 permutations was used. Percent tree cover was tested at 50‐m intervals at radii from 100 m to 1000 m.

To test whether the composition of bat and fly assemblages were correlated, a Mantel test using 10,000 permutations and the Pearson's product‐moment were used to estimate the correlation between the two Chao dissimilarity matrices. The assemblage data were visualized using nonmetric multidimensional scaling (NMDS) plots. All assemblage analyses were carried out using the “vegan” package (Oksanen et al. 2015).

Because male bats hosted fewer parasites than female bats (see Results), relationships between male bat assemblages and their parasite assemblages may not be detected due to lack of power. To account for this, we randomly selected as many parasites as were found on male bats from female bats. We then determined the correlation between these random assemblages and the female bat assemblages. This was repeated 100 times.

Because our goal was to compare between sites, we tested whether the assemblages of bats at each site in a given year were more similar to the assemblage of bats at the same site in the other year or nearby sites in the same year. Consistent assemblages across years in a given site that are not overly similar to neighboring sites may indicate that species assemblages were adequately sampled and the sampling was of effective scale. Mantel tests were used as above to examine the correlation between the Chao dissimilarity matrices of the bat assemblages in different years and a matrix of Euclidean distances between sites. Additionally, we tested the correlation between the 2012 and 2013 data and capture data from 2009 through 2013 (Mendenhall et al. 2014) to ensure that the differences between sites we observed in these two years were representative and generated species accumulation curves to determine whether we had detected most of the species present in the environment.

### Model of parasite abundance on individual bats

In order to determine how sex mediates human impacts on the abundance of parasites on each bat, the effect of the following factors was tested: tree cover, species richness of bats caught at the site, host sex, and roost duration. Roost duration is an important ecological factor influencing bat fly parasitism (Patterson et al. 2007) and therefore must be included in analyses.

Bat species richness (estimated number of unique species) at each site was calculated using Chao species richness (Chao 1987), and this value was assigned to each bat at a given site as an estimate of the bat species richness it might encounter. Each bat was assigned the percentage of tree cover in a 1000 m radius around the net in which it was caught (Mendenhall et al. 2014). This scale was chosen to match the home ranges of the species in our study, because previous studies have found bat assemblage responses to tree cover at this scale (i.e., Meyer and Kalko 2008; Klingbeil and Willig 2010) and so as to minimize correlation between nearby sites. Additionally, previous work suggests that during the end of the dry season when females are pregnant, most frugivorous species (the majority of species in our study) respond to the landscape on the 1‐km scale, staying close to their roost due to the increased energetic demands of reproduction (Klingbeil and Willig 2010). The model was also tested using tree cover in a 600 m radius, the radius at which fly assemblages on male bats were most closely correlated with tree cover (Fig. S3). A species' roost duration is a rough weighted average of the durations of the substrates it typically uses; this roost duration was assigned to every individual of that species (Patterson et al. 2007). Roost durations were either taken from Patterson et al. (2007) or calculated using their formula and data from Reid (2009).

A full model with all single terms and all two‐way interactions with negative binomial error and a log link was fit. Random intercept models for species, capture year, and site, as well as a random slope model with different slopes for different species depending on tree cover, were all included independently and in all combinations, and the best random effect structure was determined using likelihood ratio tests (Appendix S1). Model selection on the best full model used a step‐down approach, dropping the least significant predictors as determined by a likelihood ratio test. Because the majority of bats lack parasites and species differ in their overall level of parasitism (see Results), we fit a zero‐inflated, generalized linear mixed effect model implemented in the glmmADMB package (Fournier et al. 2012; Skaug et al. 2014) using selection procedures adapted from Zuur et al. (2009).

## Results

### Capture summary

One thousand and sixty‐six individual bat captures were recorded, representing 36 species, from which we collected 1038 individual bat flies, representing 32 species. All flies were identified to genus and most to species. (The 18 individuals we could not identify to species were assigned to three distinct morphospecies.) Of the captured bats, 1108 individuals from 35 species were used in analyses. One species of bat, *Sturnira mordax,* totaling 36 captures, was excluded due to lack of data on its roost sites. These individuals were excluded from all analyses for consistency. The analyzed bat individuals hosted 951 bat fly individuals, representing 30 bat fly species; these flies were used in the assemblage analyses. Total number of bats captured and parasitized bats of each species, along with the number of flies and the species found on each bat species that were used in the analyses, are listed in Table S1.

The observed bat species richness in each site ranged from 5 to 23 species (13.3 ± 0.98). The Chao bat species richness in each site ranged from 6.5 to 27.3 species (16.6 ± 1.44). Neither observed species richness nor Chao species richness showed a linear relationship with tree cover at a 1000 m radius (observed species richness, *r* ^2^ = 0.050, *P* = 0.37; Chao species richness, *r* ^2^ = 0.055, *P* = 0.35). This differs from the results of a 4‐year capture survey in this area, which found significant species richness decline outside of forest (Mendenhall et al. 2014), likely because of our smaller sample size.

Three of four bat individuals had no parasites (833 individuals), with species‐specific prevalence of parasitized individuals varying from 0% to 100%. The average prevalence per species was 32.0% (SE ± 5.8%). Of the analyzed bats, 536 were female and 572 were male; 165 of the females were parasitized while 110 of the males were parasitized. Females were more frequently parasitized in 14 of the 26 species for which we captured male and female individuals (female prevalence/male prevalence: 1.89 ± 0.15; average excludes one species in which males were not parasitized); males were more frequently parasitized in 7 (male prevalence/female prevalence: 1.66 ± 0.47; average excludes two species in which females were not parasitized). Parasitized females had an average of 4.13 parasites (SE ± 0.70) while parasitized males had 2.99 parasites (SE ± 0.32). Of the 20 species with parasitized females and males, parasitized females hosted more parasites than males in 12 species (4.00 ± 2.39 more parasites on female individuals than male individuals), while males hosted more parasites in 6 (0.89 ± 0.15). We did not detect more parasites in later sites than in earlier sites in either 2012 (Mann–Whitney *U*‐test, one‐tailed, *W* = 4947, *P* = 0.96; mean_first 4_ = 0.33, mean_last 4_ = 0.18) or 2013 (Mann–Whitney *U*‐test, one‐tailed, *W* = 8677, *P* = 0.07; mean_first 4_ = 0.86, mean_last 4_ = 0.94).

### Bat assemblage shifts

**Figure 2:**
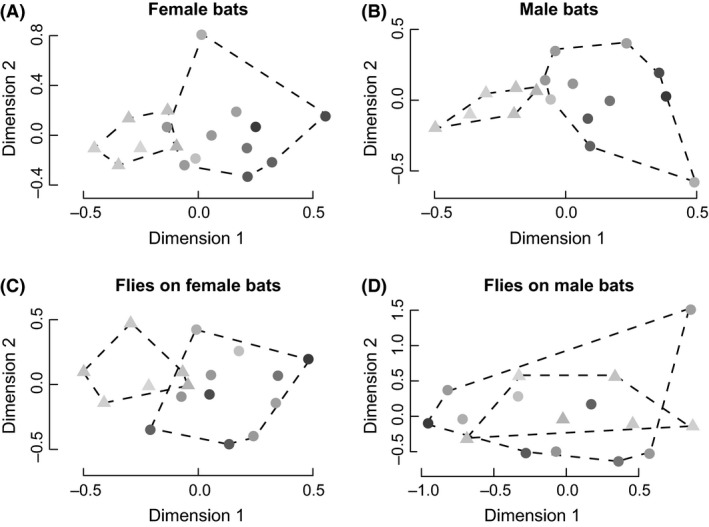
Bat assemblages (both sexes), fly assemblages on female bats, shift along a deforestation gradient; fly assemblages on males do not shift with deforestation or their host assemblages. Nonmetric multidimensional scaling plots of (A) female bats, (B) male bats, (C) flies on female bats, and (D) flies on male bats based on Chao dissimilarity indices. Circles indicate forested sites (forest reserve or forest patches). Triangles denote coffee sites. Shading indicates tree cover at 1000 m around the site; darker points indicate greater tree cover. Tree cover around sites ranged from 16.3% to 77.5%. The plots exclude one site because there were no flies collected from female bats in this site. Stresses: Female bats: 18.341; Male bats: 16.107; Flies on female bats: 16.553; Flies on male bats: 12.364.

Across all species, female and male bats were caught at each site in roughly equal numbers (paired *t*‐test, *t* = −1.095, df = 17, *P* = 0.29) and the sex ratio of all bats in a site was not correlated with tree cover (linear regression, *r* ^2^ = 0.14, *P* = 0.13), nor was it correlated with species richness (linear regression; observed species richness, *r* ^2^ = 0.006, *P* = 0.76; Chao species richness, *r* ^2^ = 0.031, *P* = 0.48). Areas with more similar tree cover hosted more similar assemblages of bats across both sexes: Both female and male bat assemblages differentiated along the tree cover gradient (Females: Fig. 2A; PERMANOVA, *F*_1,15_ = 11.101, *P* = 0.0001; Males: Fig. 2B; PERMANOVA, *F*_1,15_ = 5.286, *P* = 0.0033), and changes in the female bat assemblages were correlated with changes in the male bat assemblages (Mantel test; *r* = 0.452, *P* = 0.0017).

Bat assemblages at each site in 2012 were very similar to the bat assemblages at the same site in 2013 (Mantel test; *r* = 0.559, *P* < 0.0001; 63.5% of the most abundant five species at each site were the same between years), but bat assemblages in each site were not overly similar to bat assemblages in nearby sites (Mantel test; *r* = 0.155, *P* = 0.11), indicating adequate sampling and scale. Bat assemblages in each site during the study were also similar to bat assemblages known from 5 years of capture data (Mantel test; *r* = 0.273, *P* = 0.014; Fig. S1), and species accumulation curves are similar between the data in this study and the 5‐year dataset (Fig. S2).

### Fly assemblage shifts

Fly assemblages on female bats differed moderately between patches with different local tree cover (1000 m radius; Fig. 2C; PERMANOVA, *F*_1,15_ = 2.068, *P* = 0.055). However, the bat fly assemblages on male bats did not show the same pattern and were uncorrelated with tree cover (Fig. 2D; PERMANOVA, *F*_1,15_ = 1.547, *P* = 0.167). These relationships remained true at all radii, from 100 m to 1000 m (Fig. S3).

Changes in the composition of female bat assemblages were correlated with changes in the composition of their fly assemblages (Mantel test; *r* = 0.360, *P* = 0.0002), but this was not true of male bat assemblages and their flies (Mantel test; *r* = 0.116, *P* = 0.106). When 100 random subsets of flies off female bats were selected (the same number of flies as found on male bats), each fly assemblage subset shifted with the female bat host assemblage (Mantel test: *r*_mean_ = 0.395 [SE ± 0.0059], *p*_mean_ = 0.00083 [SE ± 1.4 × 10^−4^]; no *P* value was above 0.01). Including the site in which no flies were caught on female bats did not qualitatively change any of the observed relationships above (Appendix S2).

### Parasite load response to tree cover and bat species richness

**Figure 3:**
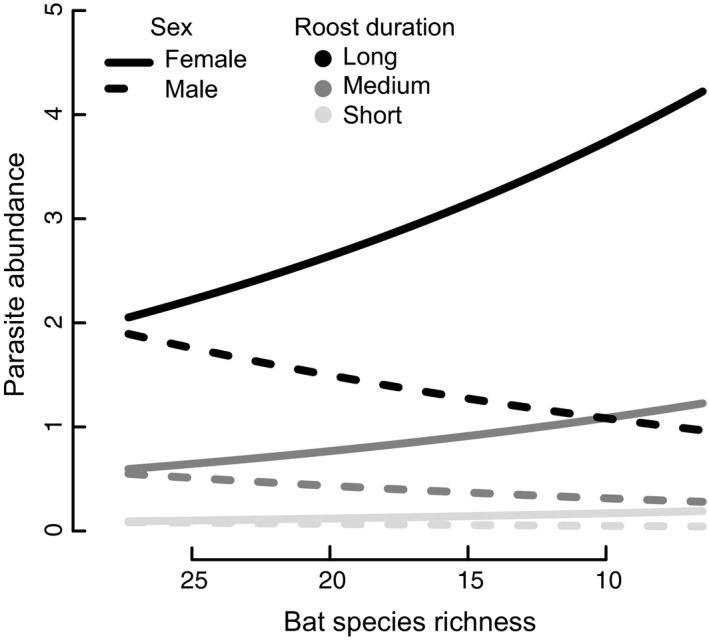
As bat species richness declines, GLMM predicts contrasting effects by sex on parasite abundance/individual. Predictions from a generalized linear mixed effect model are shown for species using roosts that last on the order of thousands of years (black curve), decades (medium gray curve), and days (light curve). We used Chao‐estimated species richness of bats in each capture site from two sampling years. The richness values in the predictions represent the actual range of richness values in the 18 sites. Note that both sexes converge on the same predicted parasite abundance/individual in very rich assemblages.

Female bats had more parasites than males and, after accounting for species identity and capture year, female bats in assemblages with high species richness hosted fewer parasites than conspecifics in comparatively species‐poor environments (*P* = 0.003). Males showed a reversal of this pattern, with males in richer assemblages hosting more parasites (Fig. 3, Table S2). Additionally, bats that use roosts of longer duration had more parasites than those that use roosts of shorter duration (*P* = 0.003). Tree cover (at 1000 m or 600 m) was not a significant predictor of parasite abundance, nor were any of the two‐way interactions significant predictors except species richness and sex. When we repeated this same analysis without sex or any of its interactions, the only significant predictor of parasitism was roost duration. Models using observed species richness for all sites yielded similar results to models using Chao species richness estimates (Table S3).

## Discussion

Our data support the hypothesis that the species of parasites present in an area and the load of parasites on an individual can be altered in a sex‐specific manner by human‐induced changes in habitat and host species richness, even if males and females are found in similar areas. These patterns were evident across the bat species in the assemblage, indicating the existence of sex‐specific responses to anthropogenic impacts that are common across species.

### Female and male bats use the landscape similarly

Our results suggest that male and female bats use the landscape in similar ways, as indicated by the similarities in male and female bat assemblages at each site, at least at the level of the presence in the various sites. However, we did observe differences in the bat species assemblages in areas of different tree cover, possibly driven by differences in available food or roosting resources preferred by different species (Medellín et al. 2000).

### Only female bats' parasite assemblages correlate with host assemblage and tree cover

Because bat fly species are highly host specific, usually being found on individuals of only one host species or a few closely related ones (Dick and Patterson 2006), we predicted that any shifts observed in bat fly assemblages would originate from shifts in bat assemblages, which in turn change with tree cover. We found support for this prediction in the fly assemblages on female bats as they changed with their host assemblages and tree cover. However, the assemblages of flies on male bats showed no apparent organization with regard to tree cover or their host assemblages (Fig. 2), indicating parasitism in males is not following the same pattern as in females. This lack of pattern is unlikely to be driven by random errors introduced by the comparatively low number of parasites on male bats because assemblages of comparably low numbers of parasites on female bats still tracked their host assemblages closely.

There are many potential explanations for the observed pattern. The close association between female bats and their parasites likely results from females generally roosting together in large numbers, in stable conditions, creating an environment in which females will be repeatedly exposed to a large population of bat fly parasites, increasing both the number of individual flies on a female bat and also the predictability of the parasite assemblage. In contrast, males frequently roost singly or in low numbers away from females and switch roosts, depending on roost availability (Morrison 1979; McCracken and Wilkinson 2000; Altringham 2011). This roost switching behavior may prevent relationships between male bat assemblages and their parasite assemblages from being as consistent as the relationships between female bats and their parasites.

The observed patterns in males could also potentially derive from differences in how bats of different sexes behave. Although we did not observe differences in the species compositions or abundances of males and females in various sites, it is possible that the males and females found in each site are roosting in very different locations. In many frugivorous lineages, males that protect harems spend most of their time defending the roost (and therefore the females) and relatively little time foraging compared to noncolony males (Altringham 2011). It is therefore possible that the majority of the males we observe are noncolony males. If these males are less able to defend roosting resources and are pushed into lower quality habitats, they may be interacting with other species of bats and parasites that they would not normally encounter, increasing the possibility of transmission of parasites between host species (Dick and Dittmar 2014).

Importantly, bat flies are not known to induce reactions in their hosts, and their fitness effects are unclear (Dick and Patterson 2006; Patterson et al. 2008), although they do appear to induce host grooming (ter Hofstede and Fenton 2005) and individual bat flies in this study have been found to host *Bartonella* and share strains with their bat host individuals (Judson et al. 2015). And, as blood‐sucking organisms that can move between host individuals when bats are in close contact (Reckardt and Kerth 2009), bat flies could potentially transmit disease organisms between bat individuals (Dick and Dittmar 2014). Because bat flies are so host specific, impacts of changes in fly assemblage dynamics will likely only affect the host species, unlike in the case of the more generalized flea vectors. Still, these results demonstrate that, when considering how parasite‐mediated disease risk for bats may change in this landscape, a focus on females might yield the greatest insights, as they seem to strongly affect the parasite assemblages.

### Females host more parasites with increases in parasitism in less species‐rich environments, while males show the reverse pattern

We found that females are generally more heavily parasitized than males. Female bats of many species, including Neotropical species, show heavier parasitism according to a number of metrics (Christe et al. 2007; Patterson et al. 2008). This differs from the trend usually observed in mammals in which males are more heavily parasitized (Zuk and McKean 1996), and may be caused by the aggregation of females and their young in close proximity, which provides opportunities for horizontal and vertical transmission (Christe et al. 2007; Patterson et al. 2008).

We also observed an increase in the parasite load on individual females in areas of lower bat species richness. Lower incidence of parasitism on bats in more species‐rich environments is consistent with a dilution effect as broadly defined, in which a metric of disease pressure decreases in more diverse or less disturbed habitats (Keesing et al. 2006). This decrease can be mediated by changes in host density or by the addition of less competent hosts, depending on the transmission mode of the parasite (Dobson 2004; Rudolf and Antonovics 2005; Keesing et al. 2006). As bat flies are highly host specific, others have predicted that their abundance will be affected mostly by host density (Pilosof et al. 2012). If reductions in bat species richness correlate with an increase in the density of each individual species, it could explain the pattern; areas with fewer species or poorer environments may have fewer available roosts, forcing females to crowd, increasing the probability of individuals encountering host‐specific parasites, and causing parasite abundance to increase.

Alternatively or additionally, bat fly abundance in each species may be influenced by the presence of bats of other species, not just the indirect effect of additional species decreasing host species density. Many of the bats caught, for example, *Anoura geoffroyi*,* Glossophaga soricina,* and *Carollia perspicillata*, roost with other species (Wynne and Pleytez 2005; Ortega and Alarcón‐D 2008; Altringham 2011). Relatively few bat fly species infest multiple host species (Dick and Patterson 2006) and among the bat fly species in our study that infested multiple species one bat species usually hosted the majority of fly individuals. For example, the bat fly *Anastrebla modestini* was found on *A. geoffroyi* and *Lonchophylla robusta*, bat species that sometimes roost together (Ortega and Alarcón‐D 2008), but eight times as many *A. modestini* individuals were found on *A. geoffroyi* bat individuals as on *L. robusta* bat individuals. In this case, each nonhost species of bat essentially serves as a less competent bat fly host, reducing both the proportion of infested individuals and the encounters between infested and susceptible target hosts (Dobson 2004; Rudolf and Antonovics 2005; Keesing et al. 2006). If the fly settles on a nonhost bat species, it may be less likely to survive due to host immune or behavioral responses (Dick and Patterson 2007) and individual parasitism will decrease.

Additionally, because large colonies require significant resources and lactating females often forage close to their roosts (Kunz and Lumsden 2003), areas with the greatest roosting and foraging resources will likely also support the most species. Females may also use resource poor areas, potentially indicated by lower species richness; indeed, bats have been found with offspring in anthropogenic habitats in this landscape (Mendenhall et al. 2014). However, females in resource poor areas may be more stressed than their counterparts in richer areas (e.g., Chapman et al. 2006). These stressed individuals may be worse at eliminating parasites through behavioral or immune responses (Zuk and McKean 1996), thereby increasing their parasite load regardless of whether they are roosting with other species. Because lactating females tend to forage close to their roosts (Kunz and Lumsden 2003) and the bat species included in our study tend to maintain home ranges that roughly match the scale of our landscape, species richness in a capture site is probably a reasonably accurate proxy for the species richness of bats roosting in the area and, potentially, for interspecific interaction.

In contrast to the observed reduction in parasitism on females, individual males had a higher parasite load in areas of higher species richness. This pattern may derive from differences in roost availability and competition. Given interspecific competition for predator‐free roost sites (Kunz and Lumsden 2003), there may be more competition for roosts between males in habitats with more bat species. In general, bats are more faithful to their roosts when roost availability is low (Brigham 1991; Lewis 1995; Chaverri et al. 2007); in this case, intense roost competition may cause males to be more faithful to their roosts in areas with higher species richness where there are fewer available roosts, in essence increasing their roost duration and their parasite burden.

As we did not directly observe roosts, we cannot determine which of these possible explanations is driving the observed pattern. It is likely a combination of these factors, as well as other factors that we were unable to consider. The patterns we observe are consistent with an aggregate pattern in which females roost in larger, more stable groups than most males during the maternity season. There is considerable heterogeneity in bat roosting habits, which undoubtedly introduces noise into the search for patterns across multiple bat species. However, seasonal sexual segregation is common amongst bats and is likely to be common in Phyllostomidae (Fleming 1988), the family that dominates our study (29 of 35 species). Of the 16 species for which we could find information on the roosting habits of the sexes, 12 either form maternity colonies and/or are found in single male, multifemale groups (Table S4). Therefore, the patterns we observe can be interpreted assuming most females are roosting together and away from most males, even though individual species may differ from this pattern.

Interestingly, tree cover was not correlated with the abundance of parasites on individual bats. Bat abundances are known to respond to a variety of landscape features including physical structure and configuration (Meyer and Kalko 2008; Klingbeil and Willig 2010; Cisneros et al. 2015). As our goal was to assess two commonly invoked metrics of disturbance on parasite risk, we did not assess these factors but it is possible that other landscape features may affect sex‐specific parasite risk. Additionally, physical aspects of the environment may underlie and drive the observed bat species richness differences in turn affecting parasitism either directly or through their impacts on bat species richness.

The finding of a sex‐specific relationship between parasitism and anthropogenic influences raises questions about how sex‐specific behavior may affect parasitism with implications for population stability. According to our results, females are more heavily parasitized in species‐poor environments, indicating that habitat modification may threaten population survival by reducing female health, especially if bat flies are acting as reservoirs for infectious organisms. When we analyzed our data without considering sex as a factor, we found no effect of tree cover or host species richness on the parasite load of individual bats. This observed relationship might therefore provide insights for future studies of not only changes in parasitism but in disease risk with anthropogenic influence.

Numerous studies have stressed that specific features of hosts, parasites, and disease mechanisms prevent the existence of a single relationship between disease risk and human impacts to govern all systems (e.g., Salkeld et al. 2013; Young et al. 2013); however, here we show that the situation is even more complex. Studies of roosting assemblages of a single species across an anthropogenic gradient would greatly help to clarify the drivers of the pattern we observed by investigating roosting group densities, roost fidelity, stress, and interspecific interaction across the landscape and how these factors vary with sex. However, we recognize that such studies will be logistically complex, especially given the difficulties of radio tracking (e.g., Fenton et al. 2000) and discovering roosts, especially small nonmaternity roosts.

## Conclusions

Understanding the interplay between parasite risk, habitat change, and host sex will become more important for conservation and public health as humans further intensify global change. Countryside landscapes, such as the one in which we worked, are the types of environments where humans are most likely to come into contact with wildlife, leading to disease sharing between species (Karesh et al. 2012). It is therefore critical that further research is devoted to examining how this extensive landscape type affects parasite risk in bats, considered to be the reservoir for some of the most lethal emerging zoonoses (De Luis et al. 2013).

More broadly, the failure to consider sex differences may partially account for the heterogeneity in the observed relationships (or lack thereof) between wildlife disease and anthropogenic activities. Additionally, the sex‐specific trend across species, likely due to shared behaviors across species within sexes, indicates that in some cases it may be appropriate to test the effect of ecological stressors on sexes in addition to species. Indeed, demonstration that sexes behave similarly across species may be a powerful way to consider interactions between landscape change and survival of bats, which are powerful ecosystem service providers (Kunz et al. 2011).

## Data Accessibility

The datasets supporting this article have been uploaded as supplementary material.

## Conflict of Interest

None declared.

## Supporting information


**Figure S1.** Assemblage change across sites in the study years is similar to assemblage change across sites in a 5 year capture dataset.
**Figure S2**. Bat species accumulation curves show adequate sampling of most species.
**Figure S3.** Correlation of bat and bat fly communities with tree cover at different spatial scales.
**Table S1.** Bat and bat fly summary of individuals used in models and community analyses.
**Table S2.** Summary of best model.
**Table S3.** Summary of best model with observed species richness.
**Table S4.** Roosting habits of select species contained within the study.
**Appendix S1.** Model selection for model family and random effects for the full model.
**Appendix S2.** Results of community analyses including excluded site.

**Appendix S3.** Data on bat captures, bat and bat fly identities and tree cover at each site.
