# Supplementary material for: Anthropogenic impacts on Costa Rican bat parasitism are sex specific
Source: Ecol Evol. 2016 Jun 21;6(14):4898–909. doi: 10.1002/ece3.2245 (PMC4979715; doi:10.1002/ece3.2245)
Supplement: Supplementary file 1 — Figure S1. Assemblage change across sites in the study years is similar to assemblage change across sites in a 5 year capture dataset. Figure S2. Bat species accumulation curves show adequate sampling of most species. Figure S3. Correlation of bat and bat fly communities with tree cover at different spatial scales. Table S1. Bat and bat fly summary of individuals used in models and community analyses. Table S2. Summary of best model. Table S3. Summary of best model with observed species richness. Table S4. Roosting habits of select species contained within the study. Appendix S1. Model selection for model family and random effects for the full model. Appendix S2. Results of community analyses including excluded site. [file ECE3-6-4898-s001.docx]

Supporting Information for:

**Anthropogenic impacts on Costa Rican bat parasitism are sex-specific**

Hannah K. Frank, Chase D. Mendenhall, Seth D. Judson, Gretchen C. Daily and Elizabeth A. Hadly

This file contains:

**Figure S1:** Assemblage change across sites in the study years is similar to assemblage change across sites in a five year capture dataset

**Figure S2**: Bat species accumulation curves show adequate sampling of most species.

**Figure S3:** Correlation of bat and bat fly communities with tree cover at different spatial scales

**Table S1:** Bat and bat fly summary of individuals used in models and community analyses

**Table S2:** Summary of best model

**Table S3:** Summary of best model with observed species richness

**Table S4:** Roosting habits of select species contained within the study

**Appendix S1:** Model selection for model family and random effects for the full model

**Appendix S2:** Results of community analyses including excluded site

**References cited in the Supporting Information**

“**Appendix S3:** Data on bat captures, bat and bat fly identities and tree cover at each site” is available as a separate .xls spreadsheet.

**Figure S1:** *Assemblage change across sites in the study years is similar to assemblage change across sites in a five year capture dataset*

Nonmetric multidimensional scaling plots of bat assemblages in each site in the study years (left) versus in a larger dataset from this landscape (right) based on Chao dissimilarity indices. Triangles denote non-forest sites (coffee plantations). Circles indicate forested sites (forest reserve or forest patches). Shading indicates tree cover at 1000m around the site; darker points indicate greater tree cover. Tree cover around sites ranged from 16.3% to 77.5%. Stresses: 2012-2013 data: 16.23; 2009-2013 data: 17.45

**Figure S2**: *Bat species accumulation curves show adequate sampling of most species*.

Species accumulation curves for all of the coffee sites (left), forest sites (middle) or all sites combined (right). Blue curves represent bat species accumulation in five years worth of data from these sites. Green curves represent bat species accumulation in the two study years. Pink curves represent bat species accumulation of common species (i.e. only of species which make up over 1% of the total abundance) in five years of capture data. Yellow curves represent species accumulation of these same common species during the study years. Note that in the forest sites and combined data the yellow is largely obscuring the pink due to their close alignment.

**Figure S3:** *Correlation of bat and bat fly communities with tree cover at different spatial scales*.

P values correspond to the results of PERMANOVA tests examining the relationship between animal communities and tree cover at various radii, on the x-axis, at the site in which the bat was captured. Distances are in meters. Black lines correspond to females; gray lines correspond to males. Dashed lines indicate fly communities on bats of a given sex, denoted by color; solid lines indicate bat communities of each sex, denoted by color.

| **Species** | **N** | **Bat fly species found on bat species** | **N** |
| --- | --- | --- | --- |
| *Anoura cultrata* | 2 (0) | N/A |  |
| *Anoura geoffroyi* | 30 (27) | *Anastrebla modestini, Exastinion clovisi* | 32/ 150 |
| *Artibeus jamaicensis* | 90 (15) | *Aspidoptera phyllostomatis, Megistopoda aranea* | 15/ 9 |
| *Artibeus lituratus* | 77 (21) | *Neotrichobius* new spp. #1^†^*, Paratrichobius longicrus* | 2/ 44 |
| *Artibeus phaeotis/watsoni* ^*^ | 109 (1) | N/A |  |
| *Artibeus watsoni* | 72 (0) | N/A |  |
| *Carollia castanea* | 111 (36) | *Strebla guajiro, Trichobius joblingi* | 4/ 56 |
| *Carollia perspicillata* | 73 (14) | *Speiseria ambigua, Strebla guajiro, Trichobius joblingi* | 3/ 1/ 26 |
| *Carollia sowelli* | 42 (13) | *Strebla guajiro, Trichobius joblingi* | 1/ 17 |
| *Chiroderma salvini* | 1 (0) | N/A |  |
| *Desmodus rotundus* | 10 (2) | *Trichobius parasiticus* | 3 |
| *Enchisthenes hartii* | 4 (2) | *Paratrichobius longicrus* | 2 |
| *Eptesicus brasiliensis* | 2 (1) | *Anatrichobius scorzai* | 1 |
| *Glossophaga commissarisi* | 21 (4) | *Anastrebla nycteridis, Strebla guajiro, Trichobius joblingi* | 1/ 1/ 1 |
| *Glossophaga soricina* | 74 (5) | *Anastreba mattadeni, Strebla guajiro, Trichobius dugesii* | 8/ 1/ 1 |
| *Hylonycteris underwoodi* | 18 (8) | *Exastinion clovisi, Strebla* new species #1^†^ | 11/ 13 |
| *Lonchophylla robusta* | 28 (26) | *Anastrebla modestini, Anastrebla nycteridis, Trichobius lonchophyllae* | 4/ 12/ 146 |
| *Micronycteris hirsuta* | 2 (0) | N/A |  |
| *Micronycteris microtus* | 6 (2) | *Trichobius keenani* | 3 |
| *Micronycteris minuta* | 1 (0) | N/A |  |
| *Micronycteris schmidtorum* | 1 (0) | N/A |  |
| *Myotis keaysi* | 23 (12) | *Anatrichobius scorzai, Basilia* spp.^‡^ (Nycterbiidae) | 9/ 5 |
| *Myotis nigricans* | 1 (1) | *Anatrichobius scorzai* | 1 |
| *Myotis riparius* | 1 (0) | N/A |  |
| *Phyllostomus discolor* | 7 (6) | *Trichobius costalimai, Trichoboides perspicillatus,* | 9/ 38 |
| *Phyllostomus hastatus* | 4 (4) | *Strebla hertigi* | 12 |
| *Platyrrhinus helleri* | 35 (0) | N/A |  |
| *Platyrrhinus vittatus* | 28 (12) | *Paratrichobius longicrus, Trichobius* spp.^§^ | 11/ 11 |
| *Pteronotus parnellii* | 8 (8) | *Nycterophilia parnelli, Trichobius caecus, Trichobius costalimai, Trichobius johnsonae* | 18/ 166/ 8/ 13 |
| *Sturnira parvidens* | 113 (47) | *Aspidoptera delatorrei, Megistopoda proxima* | 21/ 50 |
| *Sturnira hondurensis* | 2 (1) | *Megistopoda proxima* | 3 |
| *Thyroptera tricolor* | 1 (0) | N/A |  |
| *Uroderma bilobatum* | 74 (4) | *Paratrichobius dunni* | 4 |
| *Vampyrum spectrum* | 1 (0) | N/A |  |
| *Vampyressa thyone* | 36 (3) | *Neotrichobius* spp., *Trichobius* spp. | 2/ 2 |

**Table S1:** *Bat and bat fly summary of individuals used in models and community analyses.*

“N” next to the bats denotes the number of individuals of that species used in the analyses. The number in parentheses indicates the number of parasitized individuals. “N” next to the bat fly species denotes the number of collected individuals of each bat fly species.

NB: Not all observed bat flies were captured.

^*^*Artibeus phaeotis* and *A. watsoni* are difficult to distinguish (see main text).

^†^ As described by Miller & Tschapka (2009)

^‡^ Possibly *Basilia myotis*

^§^ Possibly *Trichobius vampyropsis*

| Parasites ~ Duration + Richness + Sex + Richness: Sex, link = log | | | | | |  |
| --- | --- | --- | --- | --- | --- | --- |
| Variable | Est. Coef. | Est. SE | AIC | LRT | p | |
| Duration | 0.618 | 0.192 | 1934.3 | 8.904 | 0.0028 | |
| Richness | -0.035 | 0.016 | - | - | - | |
| Sex (male) | -1.909 | 0.444 | - | - | - | |
| Richness: Sex (male) | 0.067 | 0.023 | 1934.1 | 8.630 | 0.0033 | |
| Intercept | -2.043 | 0.801 |  |  |  | |
| Random effects | Est. Var. |  |  |  |  | |
| Species | 2.458 |  |  |  |  | |
| Year | 0.113 |  |  |  |  | |
| Model parameters | Estimate | SE |  |  |  | |
| Negative binomial dispersion parameter | 0.841 | 0.121 |  |  |  | |
| Zero-inflation | 10^-6^ | 2.10 x 10^-8^ |  |  |  | |
| Log-likelihood | -954.72 |  |  |  |  | |
| AIC | 1927.4 |  |  |  |  | |

**Table S2:** *Summary of best model*. The best-fit model of the number of parasites on an individual bat was a zero-inflated negative binomial model with random intercepts for sampling year and species of bat. The richness used in this model was Chao species richness except in cases where the result was obtuse. (See methods.)

| Parasites ~ Duration + Richness (Observed) + Sex + Richness (Observed): Sex, link = log | | | | | | |
| --- | --- | --- | --- | --- | --- | --- |
| Variable | Est. Coef. | Est. SE | AIC | | LRT | p |
| Duration | 0.621 | 0.192 | 1932.1 | | 8.926 | 0.0028 |
| Richness (observed) | -0.065 | 0.022 | - | | - | - |
| Sex (male) | -1.992 | 0.458 | - | | - | - |
| Richness (observed): Sex (male) | 0.0869 | 0.029 | 1932.5 | | 9.258 | 0.0023 |
| Intercept | -1.696 | 0.818 |  | |  |  |
| Random effects | Est. Var. |  |  |  | |  |
| Species | 2.484 |  |  |  | |  |
| Year | 0.114 |  |  |  | |  |
| Model parameters | Estimate | SE |  |  | |  |
| Negative binomial dispersion parameter | 0.851 | 0.122 |  |  | |  |
| Zero-inflation | 10^-6^ | 7.66 x 10^-9^ |  |  | |  |
| Log-likelihood | -953.60 |  |  |  | |  |
| AIC | 1925.2 |  |  |  | |  |

**Table S3:** *Summary of best model with observed species richness.* When observed species richness is used in the best model (Table S2), the resulting model is qualitatively similar.

| **Species** | **Forms maternity colonies?** | **Female roosting habits** | **Male roosting habits** | **References** |
| --- | --- | --- | --- | --- |
| *Anoura geoffroyi* | Yes | Seasonal maternity colonies; mixed sex colonies | Seasonal segregation; mixed sex colonies | (Eisenberg 1989; Galindo-Galindo *et al.* 2000) |
| *Artibeus jamaicensis* | No | Year round harems | Harem males and males roosting singly or in small groups | (Morrison 1979; Fleming 1988; McCracken & Wilkinson 2000; Reid 2009) |
| *Artibeus lituratus* |  | Year round harems with loose female associations and offspring in the colony | Harem males and some roosting singly | (Muñoz-Romo, Herrera & Kunz 2008; Reid 2009) |
| *Artibeus watsoni* |  | Multiple females with a single male or in female only groups with or without young | Single males roosting with females; occasionally in pairs | (Chaverri, Schneider & Kunz 2008) |
| *Carollia perspicillata* | Sometimes | Harems; female clusters within larger roost | Harem males or bachelor male cluster | (Fleming 1988; McCracken & Wilkinson 2000) |
| *Desmodus rotundus* |  | Stable female groups of 8-12 within multi-male, multi-female groups; females in larger groups than males and forming more associations with one another | Multi-male, multi-female groups with males in smaller groups; fewer associations with males or females than females with females | (Wilkinson 1985; McCracken & Wilkinson 2000; Reid 2009) |
| *Glossophaga soricina* | Yes |  |  | (Fleming 1988; Reid 2009) |
| *Micronycteris hirsuta* | Yes |  |  | (Handley, Wilson & Gardner 1991) |
| *Myotis nigricans* |  | Large clusters of females and young, sometimes with a few males | Majority are separate from females and solitary; a few in groups with females | (Wilson & LaVal 1974) |
| *Phyllostomus discolor* |  | Live in harem groups with unstable female composition within larger colonies | Live in harem groups or small all male groups within larger colony | (Mccracken & Wilkinson 2000; Kwiecinski 2006) |
| *Phyllostomus hastatus* |  | Live in harems with stable female composition (10-100 females) with young and other lactating females | Roost in same area as females but in separate bachelor groups; single males roost with harems | (Mccracken & Wilkinson 2000; Santos *et al.* 2003) |
| *Pteronotus parnellii* | Yes (in Cuba) | Segregated from males after mating for most of the year | Segregated from females after mating | (Herd 1983; Fleming 1988; Vater *et al.* 2003) |
| *Sturnira parvidens* |  | Individually to small groups (1-10 bats) | Individual to small groups (1-3 bats) | (Fenton *et al.* 2000) |
| *Thyroptera tricolor* |  | Mixed sex colonies (1-11 bats) | Mixed sex colonies (1-11 bats) | (Findley & Wilson 1974; Vonhof & Fenton 2004) |
| *Uroderma bilobatum* | Yes | Semi-stable mostly female groups (1-16 bats) | Harem males (2-16 bats) and solitary or in groups of 2-3 | (Lewis 1992; Mccracken & Wilkinson 2000) |
| *Vampyrum spectrum* | No | In M-F pair with recent offspring (1-5 bats) | In M-F pair with recent offspring (1-5 bats) | (Vehrencamp, Stiles & Bradbury 1977) |

**Table S4:** *Roosting habits of select species contained within the study*

**Appendix S1:** *Model selection for model family and random effects for the full model.*

The best models are in bold. All models include a log link and zero-inflation.

Note: The model testing the effect of year as the sole random effect failed to converge and is not reported.

**Comparison of family: Poisson versus negative binomial**

Fixed effects: Parasites ~ Forest cover + Roost Duration + Richness + Sex + Forest cover: Duration + Forest cover: Richness + Forest cover: Sex + Duration: Richness + Duration: Sex+ Richness+ Sex; log link

| Model 1 | Random: None | | | Family: Poisson | |
| --- | --- | --- | --- | --- | --- |
| Model 2 | Random: None | | | Family: Negative binomial | |
| Model | # parameters | Log likelihood | df | Deviance | P (>Chi) |
| 1 | 12 | -1197.2 |  |  |  |
| **2** | **13** | **-1072.9** | 1 | 248.66 | <10^-15^ |

**Comparison of random intercept: No random effect versus random intercept by species**

Fixed effects: Parasites ~ Forest cover + Roost Duration + Richness + Sex + Forest cover: Duration + Forest cover: Richness + Forest cover: Sex + Duration: Richness + Duration: Sex+ Richness+ Sex

| Model 1 | Random: None | | | Family: Negative binomial | |
| --- | --- | --- | --- | --- | --- |
| Model 2 | Random: ~ (1\|Species) | | | Family: Negative binomial | |
| Model | # parameters | Log likelihood | df | Deviance | P (>Chi) |
| 1 | 13 | -1072.9 |  |  |  |
| **2** | **14** | **-958.7** | 1 | 228.44 | <10^-15^ |

**Comparison of random effects: No random effect versus random intercept by site**

Fixed effects: Parasites ~ Forest cover + Roost Duration + Richness + Sex + Forest cover: Duration + Forest cover: Richness + Forest cover: Sex + Duration: Richness + Duration: Sex+ Richness+ Sex

| Model 1 | Random: None | | | Family: Negative binomial | |
| --- | --- | --- | --- | --- | --- |
| Model 2 | Random: ~ (1\|Site) | | | Family: Negative binomial | |
| Model | # parameters | Log likelihood | df | Deviance | P (>Chi) |
| **1** | **13** | **-1072.9** |  |  |  |
| 2 | 14 | -1072.0 | 1 | 1.84 | 0.18 |

**Comparison of random effects: Random intercept by species versus random intercepts by species and site**

Fixed effects: Parasites ~ Forest cover + Roost Duration + Richness + Sex + Forest cover: Duration + Forest cover: Richness + Forest cover: Sex + Duration: Richness + Duration: Sex+ Richness+ Sex

| Model 1 | Random: ~ (1\|Species) | | | Family: Negative binomial | |
| --- | --- | --- | --- | --- | --- |
| Model 2 | Random: ~ (1\|Species) + (1\|Site) | | | Family: Negative binomial | |
| Model | # parameters | Log likelihood | df | Deviance | P (>Chi) |
| **1** | **14** | **-958.7** |  |  |  |
| 2 | 15 | -958.2 | 1 | 0.94 | 0.33 |

**Comparison of random effects: Random intercept by species versus random intercepts by species and year**

Fixed effects: Parasites ~ Forest cover + Roost Duration + Richness + Sex + Forest cover: Duration + Forest cover: Richness + Forest cover: Sex + Duration: Richness + Duration: Sex+ Richness+ Sex

| Model 1 | Random: ~ (1\|Species) | | | Family: Negative binomial | |
| --- | --- | --- | --- | --- | --- |
| Model 2 | Random: ~ (1\|Species) + (1\|Year) | | | Family: Negative binomial | |
| Model | # parameters | Log likelihood | df | Deviance | P (>Chi) |
| 1 | 14 | -958.7 |  |  |  |
| **2** | **15** | **-952.4** | 1 | 12.56 | 0.00039 |

**Comparison of random effects: Random intercept by species and year versus random intercepts by species, year and site**

Fixed effects: Parasites ~ Forest cover + Roost Duration + Richness + Sex + Forest cover: Duration + Forest cover: Richness + Forest cover: Sex + Duration: Richness + Duration: Sex+ Richness+ Sex

| Model 1 | Random: ~ (1\|Species) + (1\|Year) | | | Family: Negative binomial | |
| --- | --- | --- | --- | --- | --- |
| Model 2 | Random: ~ (1\|Species) + (1\|Year) + (1\|Site) | | | Family: Negative binomial | |
| Model | # parameters | Log likelihood | df | Deviance | P (>Chi) |
| **1** | **15** | **-952.4** |  |  |  |
| 2 | 16 | -951.8 | 1 | 1.33 | 0.25 |

**Comparison of random effects: Random intercept by species and year versus random intercepts by species and year plus a random slope with forest cover by species**

Fixed effects: Parasites ~ Forest cover + Roost Duration + Richness + Sex + Forest cover: Duration + Forest cover: Richness + Forest cover: Sex + Duration: Richness + Duration: Sex+ Richness+ Sex

| Model 1 | Random: ~ (1\|Species) + (1\|Year) | | | Family: Negative binomial | |
| --- | --- | --- | --- | --- | --- |
| Model 2 | Random: ~ (1\|Species) + (1\|Year) + (0 + Forest cover\| Species) | | | Family: Negative binomial | |
| Model | # parameters | Log likelihood | df | Deviance | P (>Chi) |
| **1** | **15** | **-952.38** |  |  |  |
| 2 | 16 | -951.78 | 1 | 1.20 | 0.27 |

**Comparison of random effects: Random intercepts by year plus a random slope with forest cover by species versus random intercepts by species and year plus a random slope with forest cover by species**

Fixed effects: Parasites ~ Forest cover + Roost Duration + Richness + Sex + Forest cover: Duration + Forest cover: Richness + Forest cover: Sex + Duration: Richness + Duration: Sex+ Richness+ Sex

| Model 1 | Random: ~ (1\|Year) + (0 + Forest cover\| Species) | | | Family: Negative binomial | |
| --- | --- | --- | --- | --- | --- |
| Model 2 | Random: ~ (1\|Species) + (1\|Year) + (0 + Forest cover\| Species) | | | Family: Negative binomial | |
| Model | # parameters | Log likelihood | df | Deviance | P (>Chi) |
| 1 | 14 | -970.93 |  |  |  |
| **2** | **16** | **-951.78** | 1 | 38.30 | <10^-8^ |

**Appendix S2:** *Results of community analyses including excluded site*

When we included the site in which no flies were observed on female bats, the relationships remained qualitatively similar. Both female and male bat communities shifted with tree cover at a 1000m radius (Females: PERMANOVA, F_1,16_ = 10.018, p < 0.0001; Males: PERMANOVA, F_1,16_ = 5.0378 p = 0.0049). Male and female bat communities also shifted in similar ways (Mantel test; r = 0.4058, p = 0.0019). Areas with more similar tree cover hosted more similar communities of bats across both sexes.

Fly communities on female bats underwent community shifts as local tree cover (1000m radius) declined, although the relationship was not as strong as when the site was excluded (PERMANOVA, F_1,16_ = 1.8974, p = 0.069). However the bat fly communities on male bats did not show the same pattern and were uncorrelated with tree cover (PERMANOVA, F_1,16_ = 1.8703, p = 0.0906).

The communities of female bats and their flies shifted in similar ways to one another (Mantel test; r = 0.360, p = 0.0001) but this was not true of male bat communities and their flies (Mantel test; r = 0.117, p = 0.107). When we selected 100 random subsets of flies off female bats (the same number of flies as found on male bats), each fly community subset shifted with the female bat host community (Mantel test: r_mean_ = 0.320 [SE ± 0.0058], p_mean_ = 0.0045 [SE ± 8.1 x 10^-4^]; in 99 out of 100 trials, p < 0.05; in one trial, p = 0.0512).

**References:**

Chaverri, G., Schneider, C.J. & Kunz, T.H. (2008) Mating system of the tent-making bat Artibeus watsoni (Chiroptera: Phyllostomidae). *Journal of Mammalogy*, **89**, 1361–1371.

Eisenberg, J.F. (1989) *Mammals of the Neotropics*. University of Chicago Press, Chicago.

Fenton, B.M., Vonhof, M.J., Bouchard, S., Gill, S.A., Johnston, D.S., Reid, F.A., Riskin, D.K., Standing, K.L., Taylor, J.R. & Wagner, R. (2000) Roosts Used by Sturnira lilium (Chiroptera : Phyllostomidae ). *Biotropica*, **32**, 729–733.

Findley, J.S. & Wilson, D.E. (1974) Observations on the neotropical disk-winged bat, Thyroptera tricolor spix. *Journal of mammalogy*, **55**, 562–571.

Fleming, T.H. (1988) *The Short-Tailed Fruit Bat: A Study in Plant-Animal Interactions*. University of Chicago Press, Chicago.

Galindo-Galindo, C., Castro-Campillo, A., Salame-Mendez, A. & Ramirez-Pulido, J. (2000) Reproductive events and social organization in a colony of Anoura geoffroyi (Chiroptera Phyllostomidae) from a temperate Mexican cave. *Acta Zoologica Mexicana*, 51–68.

Handley, C.O., Wilson, D.E. & Gardner, A.L. (1991) Demography and Natural History of the Common Fruit Bat, Artibeus jamaicensis, on Barro Colorado Island, Panamá. *Smithsonian Contributions to Zoology*, 1–173.

Herd, R.M. (1983) Pteronotus parnellii. *Mammalian Species*, **209**, 1–5.

Kwiecinski, G.G. (2006) Phyllostomus discolor. *Mammalian Species*, **801**, 1–11.

Lewis, S.E. (1992) Behavior of Peter’s Tent-Making Bat, Uroderma bilobatum, at Maternity Roosts in Costa Rica. *Journal of Mammalogy*, **73**, 541–546.

McCracken, G.F. & Wilkinson, G.S. (2000) Bat mating systems. *Reproductive Biology of Bats* (eds E.G. Crichton), & P.H. Krutzsch), pp. 321–362. Academic Press, San Diego, California.

Mccracken, G.E. & Wilkinson, G.S. (2000) Bat Mating Systems. *Reproductive Biology*.

Miller, J. & Tschapka, M. (2009) The Bat Flies of La Selva (Diptera: Nycteribiidae, Streblidae). URL http://www.biologie.uni-ulm.de/bio3/Batfly/index.html

Morrison, D.W. (1979) Apparent male defense of tree hollows in the fruit bat, Artibeus jamaicensis. *Journal of Mammalogy*, **60**, 11–15.

Muñoz-Romo, M., Herrera, E. a. & Kunz, T.H. (2008) Roosting behavior and group stability of the big fruit-eating bat Artibeus lituratus (Chiroptera: Phyllostomidae). *Mammalian Biology - Zeitschrift für Säugetierkunde*, **73**, 214–221.

Reid, F.A. (2009) *A Field Guide to the Mammals of Central America and Southeast Mexico*, 2nd ed. Oxford University Press, Oxford.

Santos, M., Aguirre, L.F., Vázquez, L.B. & Ortega, J. (2003) Phyllostomus hastatus. *Mammalian Species*, **722**, 1–6.

Vater, M., Kössl, M., Foeller, E., Coro, F., Mora, E. & Russell, I.J. (2003) Development of echolocation calls in the mustached bat, Pteronotus parnellii. *Journal of neurophysiology*, **90**, 2274–2290.

Vehrencamp, S.L., Stiles, F.G. & Bradbury, J.W. (1977) Observations on the foraging behavior and avian prey of the Neotropical carnivorous bat, Vampyrum spectrum. *Journal of Mammalogy*, **58**, 469–478.

Vonhof, M.J. & Fenton, M.B. (2004) Roost availability and population size of Thyroptera tricolor, a leaf-roosting bat, in north-eastern Costa Rica. *Journal of Tropical Ecology*, **20**, 291–305.

Wilkinson, G.S. (1985) The social organization of the common vampire bat - I. Pattern and cause of association. *Behavioral Ecology and Sociobiology*, **17**, 111–121.

Wilson, D.E. & LaVal, R.K. (1974) Myotis nigricans. *Mammalian Species*, **39**, 1–3.
